# An assessment of fishing communities around Lake Victoria, Uganda, as potential populations for future HIV vaccine efficacy studies: an observational cohort study

**DOI:** 10.1186/1471-2458-14-986

**Published:** 2014-09-22

**Authors:** Noah Kiwanuka, Juliet Mpendo, Annet Nalutaaya, Matthias Wambuzi, Annet Nanvubya, Paul K Kitandwe, Enoch Muyanja, Julius Ssempiira, Apolo Balyegisawa, Ali Ssetaala, for the UVRI-IAVI Research Team

**Affiliations:** Uganda Virus Research Institute-International AIDS Vaccine Initiate HIV Vaccine Program, Entebbe, Uganda; International AIDS Vaccine Initiative (IAVI), New York, USA; Department of Epidemiology and Biostatistics, College of Health Sciences, School of Public Health, Makerere University, Kampala, Uganda

**Keywords:** HIV-1 incidence, Retention, Willingness to participate, Fishing communities, Uganda

## Abstract

**Background:**

An effective HIV vaccine is still elusive. Of the 9 HIV preventive vaccine efficacy trials conducted to-date, only one reported positive results of modest efficacy. More efficacy trials need to be conducted before one or more vaccines are eventually licensed. We assessed the suitability of fishing communities in Uganda for future HIV vaccine efficacy trials.

**Methods:**

A community-based cohort study was conducted among a random sample of 2191 participants aged 18–49 years. Data were collected on socio-demographic characteristics, HIV risky behaviors, and willingness to participate in future HIV vaccine trials (WTP). Venous blood was collected for HIV serological testing. Retention/follow rates and HIV incidence rates per 100 person years at-risk (pyar) were estimated. Adjusted prevalence proportion ratios (PPRs) of retention and odds ratios (ORs) of lack of WTP were estimated using log-binomial and logistic regression models respectively.

**Results:**

Overall retention rate was 76.9% (1685/2191), highest (89%) among participants who had spent 5+ years in the community and lowest (54.1%) among those with <1 year stay. Significant predictors of retention included tribe/ethnicity, baseline HIV negative status, and longer than 1 year stay in the community. Overall WTP was 89.1% (1953/2191). Lack of WTP was significantly higher among women than men [adj.OR = 1.51 (95% CI, 1.14- 2.00)] and among participants who had stayed in fishing communities for 10 or more years relative to those with less than one year [adj.OR = 1.78 (95% CI, 1.11 - 2.88)]. Overall HIV incidence rate per 100 pyar was 3.39 (95% CI; 2.55 - 4.49). Participants aged 25–29 years had highest incidence rates (4.61 - 7.67/100 pyar) and high retention rates between 78.5 and 83.1%. In a combined analysis of retention and incidence rates participants aged 30+ years had retention rates ~80% but low incidence rates (2.45 - 3.57 per 100 pyar) while those aged 25–29 years had the highest incidence rates (4.61 - 7.67/100 pyar) and retention rates 78.5 - 83.1%.

**Conclusions:**

There is high HIV incidence, retention and WTP among fishing communities around L. Victoria, Uganda which make these communities appropriate for future HIV prevention efficacy studies including vaccine trials.

## Background

Despite the current proven methods of HIV prevention [1–10], an estimated 2.3 million people worldwide become newly infected with HIV each year. The number of new HIV infections has decreased but is still higher than the number of patients initiating Antiretroviral Therapy (ART) [11]. The development of a safe, effective, and accessible HIV preventive vaccine will greatly complement the current prevention interventions and probably lead to the ultimate control of the HIV pandemic. The discovery of an effective HIV vaccine has multiple challenges including the extensive variability of the virus, an incomplete understanding of the mechanisms for immune protection, and the need to identify and characterize appropriate populations for efficacy clinical trials. One of the critical elements of an HIV vaccine efficacy trials is the identification of populations with adequate rates of new HIV infections who are willing to participate (WTP) and who can be efficiently recruited and retained for the duration of the vaccine trial [12, 13].

To date, a few efficacy trials of HIV preventive vaccines have been conducted predominantly in highly selected populations [14–19] and all but one [20] reported lack of efficacy in protecting against HIV infection. Since an effective HIV vaccine still eludes the world, more efficacy trials will need to be conducted before licensure is attained. With a number of HIV vaccine candidates currently under phase I and II trials [21, 22], it is prudent to identify and prepare suitable populations for future efficacy trials. We assessed the suitability of fishing communities along Lake Victoria, Uganda, as a potential population for future HIV vaccine efficacy trials. In Uganda, fishing communities together with sex workers and long distance truck drivers have been shown to have high incidence rates [23–27] that are 3–7 times higher than the estimated national general population rate [28]. The high risk profile of fishing communities is attributed to a number of factors including the mobile lifestyle of fishermen [27, 29–31], high concentration of drinking and entertainment places, high levels alcohol consumption, transactional sex, multiple sexual partnerships and limited access to prevention and care services [24, 27, 32–34].

## Methods

### Study population and procedures

Between September 2011 and March 2013, we conducted a community-based cohort study among adults in 8 fishing communities (1 lakeshore and 7 islands) in 3 districts bordering Lake Victoria in central Uganda, a region that is mainly inhabited by people of Baganda tribe/ethnicity. Detailed study procedures have been previously described [35] but briefly, a random sample of 2200 participants aged 18–49 years resident in fishing communities for at least 6 months prior to baseline enrolment were selected from a community wide census database using Stata® 12 (StataCorp, College Station, TX) software. Of the 2200 selected, 2191 provided written informed consent and were enrolled at baseline. Interviewer- administered semi-structured questionnaires were used to collect data on socio-demographic characteristics, HIV risk behaviours, and willingness to participate (WTP) in future HIV vaccine studies. Over 99% of the questionnaires were administered in Luganda (the most commonly spoken language in the study communities) while others were in English. Two weeks to one month prior to the scheduled follow up visit date, community-based health education meetings were conducted, attended by both participants and non-participants. In addition to education and information sharing, participants were reminded of the impending follow up visit but no individualized contact, mailings, phone calls or written invitations were provided. At each visit, venous blood was collected for HIV-1 serological testing and participants were given free voluntary counseling and testing by certified HIV counselors. HIV infected participants were referred to HIV/AIDS care centres for further management and encouraged to seek care. Individuals in study communities (participants and non-participants) were given free access to HIV prevention services including health education, counseling, treatment of sexually transmitted infections (STIs) and voluntary medical male circumcision. After completion of the study procedures each study participant was reimbursed 5000 Uganda shillings (2 USD) for time and travel. Institutional Review Board approvals were obtained from the Uganda Virus Research Institute’s Science and Ethics Committee and the Uganda National Council for Science and Technology.

### Laboratory testing

HIV-1 status was determined by rapid HIV tests performed in the community by certified laboratory technologists and EIA confirmation in the laboratory at Uganda Virus Research Institute. In the rapid HIV testing algorithm blood samples were first tested with Determine® HIV assay (Alere Medical Co., Ltd., Chiba, Japan), and if negative, results were reported as negative. Determine® positive samples were then tested with HIV 1/2 Stat-Pak® assay (Chembio Diagnostic Systems, Inc. Medford, NY, USA), if positive, results were reported as positive. But if negative on Stat-Pak®, Uni-Gold™ HIV test (Trinity Biotech plc, Bray, Ireland) was used as a tie-breaker. All positive rapid results were confirmed using 2 parallel Enzyme Linked Immuno Sorbent Assay (EIA) tests: Vironostika (HIV Uni-Form II plus 0 microelisa system, Biomerieux, SA, Marcy l’Etoile, France); and Murex HIV-1.2.O (Diasorin S.P.A, Dartford, United Kingdom). Concordant EIA positives were taken as positive but discordant EIA results were comfirmed using HIV RNA PCR (COBAS® AmpliPrep/COBAS® TaqMan® HIV-1 Test, v2.0 from Roche Molecular Diagnostics, Pleasanton, CA, USA).

### Statistical analysis

To evaluate the suitability of fishing communities as potential populations for future HIV prevention efficacy studies, we assessed absolute HIV incidence, retention and WTP. HIV incidence rates per 100 person years at-risk (pyar) were estimated among initially seronegative participants as the number of seroconversions divided by pyar multiplied by 100. HIV infection was estimated to have occurred at the midpoint between the last negative and first positive serologic tests. Retention was calculated as the number of participants traced and interviewed at the 12 months visit divided by the number enrolled at baseline. Given the high proportion of retention/follow up (76.9%) we used log-binomial regression models to estimate unadjusted and adjusted prevalence proportion ratios (PPRs) and corresponding 95% CIs of factors associated with retention. Since odds ratios give biased estimates of prevalence ratios when the proportion of the outcome is greater than 10%, we used log binomial regression instead of logistic regression [36, 37]. The final model on retention included gender, age, tribe, occupation, marital status, and duration of stay in community. WTP was assessed hypothetically given that the study did not involve recruitment and receipt of a vaccine (experimental or licensed). This was done only at the baseline visit and the specific question on WTP was: If you were requested to participate in a research study on an experimental HIV preventive vaccine, would you be willing to join the study as a participant? The responses were willing, not willing, not sure, partner would decide and parent would decide. Only the first 3 got responses and the covariate was dichotomized into willing and not willing; not willing and not sure combined because the frequency of the latter was too small for meaningful analyses. Since we were interested in determining characteristics of fisherfolk that one would address in preparation for efficacy trials of HIV prevention, we modeled unwillingness to participate in HIV vaccine trials as the outcome. With a prevalence of 10.7%, ordinary multivariable logistic regression models were used to determine factors associated with unwillingness to participate in vaccine trials. The final model included sex, occupation, duration of stay in community, and alcohol consumption. In all multivariable models we used empirical variance estimator to estimate robust standard errors accounting for any potential correlation at household level (where more than 1 participant from a given household were selected) [38]. Inclusion of variables in multivariable models was based on biological plausibility and a bivariate statistical significance at an alpha (α) of <0.15 but statistical significance was determined by α <0.05. All regression models were constructed using stepwise logical model building method [39] and statistical analyses were performed using Stata® 12 (StataCorp, College Station, TX) software. Analyses involved participants that were HIV negative at baseline and hence at risk of infection.

## Results

### Participant retention

Table 1 shows absolute retention rates, and unadjusted and adjusted prevalence ratios (PRs) of factors associated with retention in fishing communities around Lake Victoria, Uganda. Of the 2191 participants enrolled at baseline, 1685 were interviewed at the 12 months visit giving an overall retention rate of 76.9%. The highest retention rate of 89% was seen in participants who had spent 5 or more years in fishing communities. Retention rates of 80% or more were observed in farming occupation (85.4%), Baganda tribe (80.9%), married monogamous (80.5%), HIV negative at baseline (80.2%), and being aged 30 or more years (80.6% for 30–39 years and 83.7% among 40–49 years). The lowest retention rate of 54.1% was observed among participants who at baseline had spent less than a year in fishing communities. At bivariate analysis, retention was significantly lower among participants aged 18–24 years, non-Baganda, bar/hotel/restaurant workers, single never married, baseline HIV positive, and those who had spent less than a year in the fishing communities. Retention did not statistically differ by sex, age, education status, or religion but adjusted differences were observed according to ethnicity, marital status, occupation, duration of stay in the community, and baseline HIV sero-status (Table 1). Retention was significantly higher among the Baganda ethnic group (adj.PPR = 1.07; 95% CI, 1.03 - 1.12), HIV negatives (adj.PRR = 1.19; 95% CI, 1.13 - 1.27), those not currently married but previously married (adj.PRR = 1.10; 95% CI, 1.01 - 1.18), and among those who had spent more than 1 year in the community at the time of enrolment. Compared to participants with less than 1 year of stay in the community, adjusted PRRs of retention were 1.34 (95% CI 1.21 - 1.48) for 1 to 4 years, 1.57 (95% CI, 1.43 - 1.73) for 5 to 10 years, and 1.59 (95% CI, 1.44 - 1.76) for more than 10 years of stay in fishing communities.

**Table 1:** **Proportions and prevalence proportion ratios (PPRs) of retention in a cohort of fishing communities around Lake Victoria, Uganda**

	Retention rate (12 months)	Prevalence proportion ratios of retention		
	% (No/enrolled)	Crude	Adjusted	P- value
		(95% CI)	(95% CI)	
**All Participants**	80.2 (1289/1607)			
**Sex**				
Male	79.6 (697/876)	1 (Ref)	1 (Ref)	
Female	81.0 (592/731)	1.02 (0.97 - 1.07)	1.05 (0.99 - 1.11)	0.074
**Age at enrolment** (years)				
18-24	72.8 (366/503)^††^	1 (Ref)	1 (Ref)	
25-29	79.8 (328/411)	1.10 (1.02 - 1.18)	1.00 (0.93 - 1.07)	0.933
30-39	86.1 (432/502)	1.18 (1.11 - 1.26)	1.04 (0.97 - 1.11)	0.273
40-49	85.3 (163/191)	1.17 (1.08 - 1.27)	1.00 (0.92 - 1.09)	0.953
Highest Education level*				
None	80.4 (90/112)	1 (Ref)	-	
Primary	79.4 (734/924)	0.99 (0.90 - 1.09)	-	
Post primary	81.3 (462/568)	1.01 (0.92 - 1.12)	-	
**Religion**				
Pentecostal/Evangelical	82.9 (131/158)	1 (Ref)	-	
Roman Catholic	81.6 (507/621)	0.98 (0.91 - 1.07)	-	
Protestant/Anglican	76.7 (342/446)	0.92 (0.85 - 1.01)	-	
Moslem	80.4 (263/327)	0.97 (0.89 - 1.06)	-	
Other^¶^	83.6 (46/55)	1.01 (0.88 - 1.16)	-	
**Ethnicity/tribe**				
Non-Muganda	76.9 (681/885)^††^	1 (Ref)	1 (Ref)	
Muganda	84.2 (608/722)	1.09 (1.04 - 1.15)	1.06 (1.01 - 1.11)	0.021
**Occupation**				
Fishing/Fishing related	80.5 (648/805)	1 (Ref)	1 (Ref)	
Trade/Business	81.2 (135/165)	1.02 (0.94 - 1.10)	0.96 (0.89 - 1.04)	0.382
Bar/Lodge/Restaurant	75.9 (123/162)	0.94 (0.86 - 1.03)	0.95 (0.87 - 1.05)	0.326
Farming	86.2 (75/87)	1.07 (0.98 - 1.17)	1.00 (0.92 - 1.10)	0.870
Housewife	78.3 (101/129)	0.97 (0.88 - 1.07)	0.93 (0.84 - 1.03)	0.194
Others^†^	79.9 (207/259)	0.99 (0.92 - 1.06)	0.98 (0.92 - 1.05)	0.667
**Marital status**				
Never married	70.2 (214/305)^††^	1 (Ref)	1 (Ref)	
Not currently married	79.7 (263/330)	1.14 (1.04 - 1.24)	1.03 (0.93 - 1.13)	0.570
Married monogamous	83.3 (575/690)	1.19 (1.09 - 1.29)	1.08 (0.99 - 1.18)	0.064
Married polygamous	80.4 (237/282)	1.20 (1.09 - 1.31)	1.07 (0.97 - 1.17)	0.169
**Duration in community** (years)				
Less than 1	58.3 (175/300)	1 (Ref)	1 (Ref)	
1 to 4	77.6 (464/598)	1.33 (1.20 - 1.48)	1.31 (1.17 - 1.45)	<0.0001
5 to 10	90.9 (440/484)	1.56 (1.41 - 1.72)	1.50 (1.35 - 1.66)	<0.0001
More than 10	93.3 (210/225)	1.60 (1.44 - 1.77)	1.54 (1.39 - 1.72)	<0.0001

*3 missing education, ^¶^Seventh Day Advent/Traditionist ^†^Construction/Mechanic/Government/Clerical , ^††^Statistically significant at p < 0.05 during bivariate analysis.

The overall HIV incidence rate was 3.39/100 pyar (95% CI; 2.55 - 4.49). We estimated retention rates and absolute HIV incidence rates by a combination of selected risk factors so as to assess potential characteristics that would maximize both retention and incidence in case of targeted enrollment in efficacy studies in this population (Table 2). HIV rates presented in Table 2 are absolute risks that apply to specific groups of single of combined risk factors and are not comparative. Participants aged 30 or more years tended to have high retention rates ~80% regardless of whether they were engaged in actual fishing and or alcohol drinking. In this age-group however, the HIV incidence rate ranges between 2.45 and 3.57 per 100 pyar. For every combination of risk factors, HIV incidence was highest in age group of 25–29 years, ranging between 4.61 and 7.67/100 pyar. In this same age group, retention rates between 78.5 - 83.1% were observed. For every combination of risk factors, the lowest retention rate was among 18–24 year old participants.

**Table 2:** **Retention rates and absolute HIV incidence rates by selected risk factors**

Characteristic	Retention at 12 months		Absolute HIV risk
	Percent	(Followed/enrolled)	Rate/100 pyar (95% CI)
**All participants**	76.9%	(1685/2191)	3.39 (2.55 - 4.49)
**Age at enrolment** (years)			
30+	81.5%	(822/1009)	2.45 (1.50 - 4.00)
25-29	76.7%	(434/566)	4.77 (2.96 - 7.67)
18-24	69.6%	(429/616)	3.68 (2.22 - 6.11)
**Age and fishing occupation**			
30+ and involved in fishing	81.3%	(414/509)	3.26 (1.80 - 5.90)
25-29 and involved in fishing	78.5%	(226/288)	4.61 (2.40 - 8.85)
18-24 and involved in fishing	73.4%	(177/241)	4.02 (1.92 - 8.43)
**Age and alcohol drinking**			
30+ and drinks alcohol	79.6%	(460/578)	3.34 (1.89 - 5.89)
25-29 and drinks alcohol	78.1%	(234/311)	7.67 (4.62 - 12.7)
18-24 and drinks alcohol	67.5%	(183/271)	5.67 (3.14 - 10.2)
**Age, fishing and alcohol drinking**			
30+, fishing and alcohol use	79.9%	(243/304)	3.57 (1.70 - 7.49)
25-29, fishing and alcohol use	83.1%	(138/166)	6.77 (3.39 - 13.5)
18-24, fishing and alcohol use	72.6%	(85/117)	4.99 (1.87 - 13.3)

### Willingness to participate in future HIV vaccine trials

Overall WTP was 89.1% (1953/2191) and was higher in men than women (91.2% vs 87.3%, *p* = 0.004) and among island communities relative to lakeshore ones (90.4% vs 85.8%, *p* = 0.004) (data not shown). We assessed the factors associated with lack of WTP (unwillingness to participate in future vaccine trials). As shown in Table 3, adjusted predictors of lack of WTP were sex, duration of stay in community and alcohol consumption. Lack of WTP was significantly higher among women than men [adj.OR = 1.51 (95% CI, 1.14- 2.00)] and among participants who had stayed in fishing communities for 10 or more years relative to those with less than one year [adj.OR = 1.78 (95% CI, 1.11 - 2.88)]. However, particpants who drink alcohol were less likely to lack WTP than non-drinkers [adj.OR = 0.74 (95% CI, 0.56 - 0.98)] i.e., alcohol drinkers were more willing to participate.

**Table 3:** **Multivariable analysis of factors associated with lack of willingness to participate (WTP) in hypothetical HIV vaccine trials**

	WTP		Odds ratio of lack of WTP		
	Yes	No	Crude	Adjusted	P- value
	No. (%)	No. (%)	(95% CI)	(95% CI)	
**All Participants**	1953 (89.1)	238 (10.9)			
**Sex**					
Male	1007 (91.1)	99 (8.9)^††^	1 (Ref)	1 (Ref)	
Female	946 (87.2)	139 (12.8)	1.49 (1.14 - 1.96)	1.51 (1.14 - 2.00)	0.004^††^
**Age at enrolment** (years)					
30+	896 (88.8)	113 (11.2)	1 (Ref)	-	
25-29	509 (89.9)	57 (10.1)	0.89 (0.63 - 1.24)	-	
18-24	548 (89.0)	68 (11.0)	0.98 (0.71 - 1.35)	-	
**Religion**					
Pentecostal/Evangelical	169 (85.8)	28 (14.2)^††^	1 (Ref)	-	
Roman Catholic	801 (90.0)	89 (10.0)	0.67 (0.42 - 1.06)	-	
Protestant/Anglican	546 (91.0)	54 (9.0)	0.60 (0.37 - 0.97)	-	
Moslem	361 (85.8)	60 (14.2)	1.00 (0.62 - 1.63)	-	
Other^¶^	76 (91.6)	7 (8.4)	0.55 (0.23 - 1.33)	-	
**Ethnicity/tribe**					
Non-Muganda	1080 (90.2)	117 (9.8)	1 (Ref)	-	
Muganda	873 (87.8)	121 (12.2)	1.28 (0.98 - 1.67)	-	
**Occupation**					
Fishing/Fishing related	947 (91.2)	91 (8.8)	1 (Ref)	-	
Trade/Business	196 (87.9)	27 (12.1)	1.43 (0.91 - 2.26)	-	
Bar/Lodge/Restaurant	225 (87.6)	32 (12.4)	1.48 (0.96 - 2.27)	-	
Farming	114 (87.7)	16 (12.3)	1.46 (0.83 - 2.57)	-	
Others^ŧ^	309 (87.5)	44 (12.5)	1.48 (1.01 - 2.17)	-	
Housewife	162 (85.3)	28 (14.7)	1.79 (1.14 - 2.83)	-	
**Marital status**					
Married monogamous	819 (88.7)	104 (11.3)	1 (Ref)		
Married polygamous	378 (89.4)	45 (10.6)	0.94 (0.65 - 1.36)	-	
Not currently married	450 (89.1)	55 (10.9)	0.96 (0.68 - 1.36)		
Never married	306 (90.0)	34 (10.0)	0.87 (0.58 - 1.32)	-	
**Duration in community** (years)					
Less than 1	357 (90.6)	37 (9.4)^†^	1 (Ref)	1 (Ref)	
1 to 4	737 (89.6)	86 (10.4)	1.12 (0.75 - 1.69)	1.17 (0.78 - 1.77)	0.43
5 to 10	597 (89.2)	72 (10.8)	1.16 (0.77 - 1.77)	1.26 (0.83 - 1.93)	0.28
More than 10	262 (85.9)	43 (14.1)	1.58 (0.99 - 2.53)	1.78 (1.11 - 2.88)	0.018
**Alcohol consumption**					
No	1104 (87.9)	152 (12.1)^††^	1 (Ref)	1 (Ref)	
Yes	849 (90.8)	86 (9.2)	0.70 (0.53 - 0.91)	0.74 (0.56 - 0.98)	0.04

¶Seventh Day Adventist/Traditional, ††Statistically significant at p < 0.05, ^†^Borderline statistical significance at p < 0.05, ^ŧ^Construction/Mechanic/Government/Clerical.

## Discussion

Our assessment indicates that fishing communities around Lake Victoria, Uganda, are potential populations for future HIV vaccine efficacy studies. In these communities we found an overall HIV-1 incidence rate of 3.39/100 pyar, a retention rate of 77%, and WTP of 89.3%. The overall incidence rate in this fishing community general population is about 4–5 times higher than that estimated for the national general population of Uganda [28] and 3 times higher than the rate observed in some long term cohorts in Uganda [40, 41]. Some sub-groups in these fishing communities have incidence rates as high as 7.7/100 pyar [27]. Other population groups in Uganda with comparable levels of HIV incidence include female sex workers and long distance truck drivers [23, 25, 26] which are highly selected and gender skewed groups, making it difficult to generalize findings from these populations.

Contrary to the belief that fisher folk are difficult to retain given their mobile nature, we were able to retain 77% at a 12 month inter-survey interval using passive follow up. This result is very encouraging in that with active follow up at shorter intervals such as those in a vaccine efficacy trials, retention rate is expected to be much higher than that observed in this study. Retention was higher among participants who had stayed for more than one year in the communities which may imply that recent migrants tend to be less stable and may cause challenges in follow up studies. The retention rate we found in this general population of fishing communities is consistent with that found among fisherfolk that were screened for high risk behaviours (76%) [24] and in other population-based studies in Uganda [42, 43]. In a combined analysis of retention and incidence rates participants aged 30 or more years had retention rates ~80% with incidence rates ranging between 2.45 and 3.57 per 100 pyar while those aged 25–29 years had the highest incidence rates (4.61 - 7.67/100 pyar) and high retention rates between 78.5 - 83.1%. Retention was lowest among young adults aged 18–24 years. This finding appears to indicate that among fishing communities around Lake Victoria, Uganda, maximization of both HIV incidence and retention occurs among participants aged 25–29 years. In actual vaccine efficacy trials, efficient recruitment and retention of large numbers of participants is crucial in for the success of the study [12, 13]. Our observed retention rate of 77% could be improved by methods such as frequent participant contact and active tracing, shorter follow up intervals, and use of mobile telephone technology which have improved retention in other high risk and marginalized population groups [44, 45]. However, given the unique characteristics of fishing communities it would be prudent to evaluate the effectiveness of those strategies in enhancing retention before adopting them in actual trials.

Although WTP assessed hypothetically does not necessarily reflect levels participation in a real trial [46], we found that only 10.9% of fisherfolk expressed lack of willingness to participate in future HIV vaccine trials. Lack of WTP was significantly higher among women and folks with 10 or more years of stay in communities. A similar study in Masaka district, southern Uganda, also found higher levels of lack of WTP among women than men. In that study the requirement to delay pregnancy during the trial and shortly thereafter was associated with lower levels of WTP [31]. Since both women and men will be needed to participate in future HIV vaccine efficacy trials, it is important to address issues that may lead to women's unwillingness to participate in these trials. We also found that lack of WTP was significantly lower among alcohol drinkers yet alcohol drinking is one of major risk factors for HIV infection in this population. This finding indicates that it may not be difficult to recruit "high risk" fisherfolk in future vaccine efficacy trials. However, lack of WTP was significantly higher in person who had stayed for more than 10 years in fishing communities yet retention was much better in same group. At this time we do not know the reasons for this discrepancy but plans to investigate it are underway.

The strength of this study is that it was conducted in a randomly selected general population of fishing communities. We note that the study is not without limitations. First, a long inter-survey period of 12 months does not represent the shorter follow up intervals typically seen in the early period of vaccine trials. Second, the hypothetical assessment of WTP does not necessarily reflect actual willingness observed in real vaccine trials [46]. Third, retention rates were assessed at one follow up visit (due to the design of the study) and as such represent a cross-sectional assessment at the one follow up visit.

## Conclusions

The finding of high HIV incidence, good retention rates, and high WTP, indicates that fishing communities are potential populations for HIV prevention efficacy trials including vaccines and combination interventions.

UVRI-IAVI Research Team includes Brian Kabuubi, Annie Marie Namumiina, Wangira Denis, Marion Namuleme, Polly Mukiibi, Michael Ssenkayi, Kiwagu Humphrey, Kagolo Edward, Brian Matovu, and Ali Olega.
